# Severity of Non-B and Non-C Hepatitis Versus Hepatitis B and C Associated Chronic Liver Disease: A Retrospective, Observational, Comparative Study

**DOI:** 10.7759/cureus.12294

**Published:** 2020-12-26

**Authors:** Muhammad Sohaib Asghar, Muhammad Nadeem Ahsan, Uzma Rasheed, Maira Hassan, Rumael Jawed, Marium B Abbas, Rabail Yaseen, Syed Anosh Ali Naqvi, Hera Rizvi, Mashaal Syed

**Affiliations:** 1 Internal Medicine, Dow University of Health Sciences, Karachi, PAK; 2 Nephrology, Dow University of Health Sciences, Karachi, PAK; 3 Internal Medicine, Liaquat National Hospital, Karachi, PAK; 4 Internal Medicine , Liaquat National Hospital, Karachi, PAK; 5 Internal Medicine, Dow International Medical College, Karachi, PAK; 6 General Surgery, Dow International Medical College, Karachi, PAK; 7 Gastroenterology and Hepatology, Dow International Medical College, Karachi, PAK

**Keywords:** chronic liver disease, hepatitis b infection, hepatitis c infection, chronic viral hepatitis, nonalcoholic fatty liver disease (nafld), child-pugh class, meld, severity markers, liver fibrosis, cirrhosis

## Abstract

Background and objectives

Chronic liver disease (CLD) encompasses a variety of etiologies, and the infectious causes are mainly hepatitis B and hepatitis C virus. Chronic alcohol abuse and non-alcoholic fatty liver disease also have a major contribution to CLD. The Child-Pugh scoring system indicates the probable prognosis and mortality risk of a patient with cirrhosis. The primary objective of this research is to observe the mortality risks of CLD caused by a variety of etiologies mentioned above. The secondary objective is to determine the biochemical markers that are correlating with the severity of the study groups. Another aim was to determine the Model for End-Stage Liver Disease (MELD) scoring of each study group predicting the severity of disease among the Child-Pugh classification.

Materials and methods

We broadly classified the etiologies into two study groups: (1) hepatitis B, C associated CLD (hepatitis B, C­-CLD) and (2) non-hepatitis B, C associated CLD (non-hepatitis B, C-CLD). This study was conducted as a descriptive, retrospective study involving patients admitted to the Gastroenterology Department at Dow University Hospital between July 2019 and December 2019. All patients who met the inclusion criteria were included in the study in order to document their levels of severity markers of CLD. A total of 167 individuals met the inclusion criteria, and the sampling was done through non-probability consecutive methods. All continuous variables were described as mean and standard deviations, which were then compared using an independent sample t-test. The comparison of categorical data was done either using the chi-square test or Fisher’s exact test accordingly. A p-value of <0.05 was considered statistically significant (two-tailed).

Results

The mean age of the study population was 51.83 ± 13.67, with no difference in gender and type of CLD. The frequent co-morbidities (other than CLD) found in the study population were diabetes, hypertension, ischemic heart disease, and chronic kidney disease, with most of them having significant association with non-hepatitis B, C-CLD. Both types of CLD had equal gender proportion (p=0.708). Among the study groups, 56.28% (n=94) had hepatitis B, C-CLD, out of which 18 (19%) belonged to Child-Pugh class A, 36 (38%) to Child-Pugh class B, and 40 (43%) to Child-Pugh class C, whereas 43.72% (n=71) had non-hepatitis B, C-CLD, comprising of 13% (n=10) of Child-Pugh class A patients, 42% (n=31) of Child-Pugh class B patients, and 44% (n=32) of Child-Pugh class C patients (p=0.631). Bilirubin levels (p=0.055), serum creatinine (p=0.201), and International normalized ratio (INR) are found higher in non-hepatitis B, C-CLD (p=0.312), whereas thrombocytopenia was more likely to be associated with hepatitis B, C-CLD (p=0.205). Hyponatremia was slightly associated with non-hepatitis B, C-CLD (p=0.281). The mean MELD score was comparable among the two study groups in both Child-Pugh classes A and B, but in Child-Pugh class C it was significantly higher in non-hepatitis B, C-CLD patients as compared to hepatitis B, C­-CLD (p=0.006).

Conclusion

Non-hepatitis B, C-CLD was proved to be milder in Child-Pugh class A as compared to hepatitis B, C-CLD, but its mortality risk increases with severity, as mean MELD score was found significantly higher in Child-Pugh class C. Our research was able to identify severe biochemical markers in both types of CLD.

## Introduction

Chronic liver disease (CLD) is a debilitating process that involves immedicable destruction and repair of the liver parenchyma, resulting in fibrosis and a gradual reduction in hepatic function. Cirrhosis is the penultimate stage of CLD caused by years of unidentified and unmanaged disease progression [1]. Alone in the United States of America (USA), CLD is accountable for approximately two million deaths annually, making it the ninth leading cause of mortality within the country [2,3]. Globally it is the 14th most common cause of death and the fourth most common cause of mortality among the European population. [4]. In the USA, 633,323 people suffer from cirrhosis [5]. Assessing all these excrescent numbers, we all can concur that CLD is a significant cause of mortality worldwide.

CLD encompasses a variety of etiologies, ranging from infectious to genetic to autoimmune. The autoimmune causes of CLD include autoimmune hepatitis, primary biliary cirrhosis, and primary sclerosing cholangitis. The genetic causes include hemochromatosis, Wilson’s disease, and alpha-1 antitrypsin deficiency, engaging a multitude of symptoms. Chronic biliary diseases such as recurrent bacterial cholangitis and bile duct stenosis can also lead to liver cirrhosis. The infectious causes of CLD are mainly hepatitis B virus (HBV) and hepatitis C virus (HCV). Chronic infections with these viruses are a major cause of cirrhosis globally [6]. Chronic alcohol abuse and non-alcoholic fatty liver disease also have a paramount contribution to CLD-related mortality and morbidity.

Observing the prevalence (per 100,000 people) and mortality rate (per 100,000 per year) of several CLDs through various published articles, we can confidently conclude that even though the above-mentioned causes result in a similar disease process, they differ in their incidences and prognosis. Chronic hepatitis C has a prevalence of 2,000-3,000 people and a mortality rate of 4.6. Alcoholic liver disease has the same prevalence as chronic hepatitis C and has a mortality rate of 7.2. Non-alcoholic liver disease has the highest prevalence of 6,000-35,000 and a mortality rate of 1.3-2.6. Chronic hepatitis B has a prevalence of 2,000-8,000 and a mortality rate of 0.6. The genetic causes including hemochromatosis, Wilson disease, and alpha-1 antitrypsin deficiency have a prevalence of 3.9-6,000, 3, and 20-33.3, respectively. The mortality rates of genetic causes were not available and therefore instead the percentages of globally performed liver transplantation on all patients of hemochromatosis (1%), Wilson’s disease (1.5%), and alpha-1 antitrypsin deficiency (1%) were used. Primary biliary cirrhosis, primary sclerosing cholangitis, and autoimmune hepatitis have a prevalence of 22.7-40.2, 41.5, and 16.9-42.9, respectively; lack of any concrete data on immunological cause related mortality rates of CLD resulted in the inclusion of liver transplantation percentages in primary biliary cirrhosis (12%), primary sclerosing cholangitis (8%), and autoimmune hepatitis (4%) [2].

The relevant screening to determine the etiology of cirrhosis should always be performed to predict the prognosis [7]. On the first visit to a gastroenterologist, 44% of people present with liver cirrhosis due to the subtle clinical manifestations of CLD [8]. The comparative incidences of early diagnosis of liver diseases in private gastroenterologist clinics in the USA for hepatitis C patients were 42%, much higher than 8 and 9% for alcoholic liver disease and non-alcoholic fatty liver disease, respectively, even though the latter conditions have a far higher risk of progression to cirrhosis than hepatitis C [9]. According to World Health Organization, 15 to 30% of people with chronic HCV develop cirrhosis within 20 years; on the contrary, 15-20% of people with non-alcoholic fatty liver disease develop considerable fibrosis within 10 years [10]. Without treatment, chronic HCV advances slowly in young patients until the age of 40 when things can start looking grim as the disease progresses towards advanced fibrosis. With appropriate treatment, progression to cirrhosis in chronic HCV can even be prevented; henceforth, screening of HCV is highly recommended [11-13]. Only 5% of the people affected with HBV develop chronic infection, of those 15-20% develop cirrhosis or hepatocellular carcinoma. Studies have shown that not only do anti-hepatitis B medications put a halt to the progression of hepatitis B-induced CLD but they can also assist in reverting decompensated cirrhosis to compensated cirrhosis in 74% of cases [14]. In India, alcohol-associated liver disease was the most common cause of cirrhosis, even though chronic hepatitis B had the highest incidence in general [15].

The Child-Pugh scoring system is an apparatus used to reach the probable prognosis and to assess the mortality risk of a patient with cirrhosis. It is classified into three categories: category A (5-6 points), B (7-9 points), and C (10-15 points), with A being least severe and C being the most severe category. Points are given according to the data in encephalopathy, ascites, bilirubin, albumin, and prothrombin time [16]. Cirrhosis can be classified as compensated and decompensated. Child-Pugh score holds the most essential prognostic value in decompensated cirrhosis [17]. The Model for End-Stage Liver Disease (MELD) score was developed in 2001 to predict short-term mortality risk in cirrhotic patients [18]. The primary objective of this research is to observe the mortality risks of CLD caused by a variety of etiologies mentioned previously. The secondary objective is to determine which biochemical markers are correlating with the severity of the disease progression in the study groups. We broadly classified the etiologies into two study groups: (1) hepatitis B, C associated CLD (hepatitis B, C­-CLD) and (2) non-hepatitis B, C associated CLD (non-hepatitis B, C-CLD). Child-Pugh scoring system would be our main tool for comparing the prognosis of CLD among the study groups. As discussed earlier, hepatitis B, C-CLD, though highly prevalent around the world, results in a better prognosis more often than the other causes of CLD. Another aim was to determine the MELD scoring of each study group predicting the severity of disease among the Child-Pugh classification.

## Materials and methods

This study aimed to compare the severity markers of CLD, among viral vs non-viral etiologies. It was conducted as a descriptive, retrospective study involving patients admitted to the Gastroenterology Department at Dow University Hospital between July 2019 and December 2019. All patients who met the inclusion criteria were included in the study to document the severity markers of CLD.

The study included all those patients with a diagnosis of CLD defined by the Asian Pacific Association for the Study of the Liver (APASL) [19]. The diagnoses were reaffirmed by a radiological and clinical assessment of all the patients by reviewing their medical records after taking consent from the relevant department. The age limit of patients was set at a range of 15 to 90 years. The duration of diagnosis was set at a minimum six of months or a maximum of five years prior to inclusion in the study. The study excluded all those patients who did not fulfill the criteria of APASL to be diagnosed with CLD. The study focused solely on the severity of CLD; hence, all the patients who were diagnosed with hepatocellular carcinoma through radiological imaging were excluded (34 patients). No treatment protocols were defined in the inclusion criteria of the study. A total of 167 individuals met the inclusion criteria, and the sampling was done through non-probability consecutive methods.

All analysis was conducted by using the IBM SPSS Statistics for Windows, Version 25 (IBM Corp., Armonk, NY, USA). A sample size of 167 was calculated using a Rao-soft digital sample size calculator (http://www.raosoft.com/samplesize.html) in which we used 5% as a margin of error, 95% as confidence interval (CI), 300 as population size, and response distribution as 50%. All continuous variables were described as mean and standard deviations, which were then compared using an independent sample t-test. The comparison of categorical data was done either using the chi-square test or Fisher’s exact test accordingly. A p-value of <0.05 was considered statistically significant (two-tailed).

## Results

The mean age of the study population was 51.83 ± 13.67, with no difference in gender and type of CLD (viral vs. non-viral). The majority of the patients belonged to the age group of more than 50 years (p<0.001). The study had 54.5% males (n=91), of whom 50 were suffering from hepatitis B, C-CLD, whereas the rest 41 were diagnosed with non-hepatitis B, C-CLD. There were 76 female participants (45.5%), of whom 44 had hepatitis B, C-CLD, whereas 32 were suffering from non-hepatitis B, C-CLD. The frequent co-morbidities (other than CLD) found in the study population included diabetes, hypertension, ischemic heart disease, and chronic kidney disease. Most of them had a significant association with non-hepatitis B, C-CLD as compared to virus-associated CLD, as shown in Table 1.

**Table 1:** Demographic data of the study population (n=167). *Indicates Fisher’s exact test. †Indicates chi-square test. **Indicates independent sample t-test. CLD, chronic liver disease; IHD, ischemic heart disease; CVA, cerebrovascular accident; HIV, human immunodeficiency virus; CKD, chronic kidney disease; TB, tuberculosis

Variables	All study participants (n=167)	Hepatitis B, C- CLD (n=94)	Non-hepatitis B, C-CLD (n=73)	p-Value
Mean age (in years)	51.83 ± 13.67	51.50 ± 14.27	52.08 ± 13.25	0.787*
Gender				
Males	n=91 (54.5%)	50 (53.2%)	41 (56.2%)	0.702^†^
Females	n=76 (45.5%)	44 (46.8%)	32 (43.8%)	
Co-morbidities (other than CLD)				
Diabetes mellitus	40.11% (n=67)	28.72% (n=27)	54.79% (n=40)	0.001^†^
Hypertension	22.75% (n=38)	15.95% (n=15)	31.50% (n=23)	0.017^†^
IHD	13.77% (n=23)	9.71% (n=11)	16.43% (n=12)	0.378^†^
CVA	6.58% (n=11)	4.25% (n=4)	9.58% (n=7)	0.214*
Autoimmune	4.19% (n=7)	1.06% (n=1)	8.21% (n=6)	0.044*
Wilson’s disease	0.59% (n=1)	0.0% (n=0)	1.36% (n=1)	0.437*
HIV	1.19% (n=2)	1.06% (n=1)	1.36% (n=1)	1.000**
CKD	11.37% (n=19)	7.44% (n=7)	16.43% (n=12)	0.070^†^
TB	1.79% (n=3)	2.12% (n=2)	1.36% (n=1)	1.000*
Hypothyroidism	5.38% (n=9)	2.12% (n=2)	9.58% (n=7)	0.043*
Viral hepatitis	56.28% (n=94)	Hepatitis B: 13.77% (n=23); hepatitis C: 42.51% (n=71)	-	-
Child-Pugh’s class				
Class A	28 (16.76%)	18 (19.1%)	10 (13.7%)	0.631^†^
Class B	67 (40.11%)	36 (38.3%)	31 (42.5%)	
Class C	72 (43.11%)	40 (42.6%)	32 (43.8%)	

Among the study groups, 56.28% (n=94) had hepatitis B, C-CLD, of whom 18 (19%) belonged to Child-Pugh class A, 36 (38%) to Child-Pugh class B, and 40 (43%) to Child-Pugh class C, whereas 43.72% (n=71) had non-hepatitis B, C-CLD, comprising of 13% (n=10) of Child-Pugh class A, 42% (n=31) of Child-Pugh class B, and 44% (n=32) of Child-Pugh class C patients (p=0.631). Table 2 shows baseline laboratory investigations and comparisons among the study groups. Bilirubin levels (p=0.055), serum creatinine (p=0.201), and international normalized ratio (INR) are likely to be higher in non-hepatitis B, C-CLD (p=0.312), whereas thrombocytopenia was more likely to be associated with hepatitis B, C-CLD (p=0.205). Hyponatremia was comparatively increased in non-hepatitis B, C-CLD (p=0.281), whereas mean MELD scores were also found higher in non-hepatitis B, C-CLD patients (p=0.109). Among Child-Pugh classes, most of the aforementioned laboratory investigations were correlating with disease severity, apart from alanine transaminase (ALT) and serum creatinine, which were found slightly more elevated in Child-Pugh class B as compared to class C.

**Table 2:** Comparison of biochemical markers among patients with chronic liver disease. All the p-values were calculated using an independent sample t-test. CLD, chronic liver disease; INR, international normalized ratio; MELD, model for end-stage liver disease

Laboratory investigations	All patients (n=167)	Grouping variables		p-Value
		Hepatitis B, C-CLD (n=94)	Non-hepatitis B, C-CLD (n=73)	
Total bilirubin (mg/dL)	3.62 ± 5.48	2.90 ± 3.01	4.54 ± 7.49	0.055
Direct bilirubin (mg/dL)	2.00 ± 3.24	1.62 ± 1.86	2.49 ± 4.39	0.086
Indirect bilirubin (mg/dL)	1.65 ± 2.35	1.33 ± 1.34	2.05 ± 3.18	0.052
INR	1.88 ± 1.77	1.76 ± 1.74	2.04 ± 1.80	0.312
Platelet counts (10^9^/L)	121.87 ± 82.64	114.72 ± 86.70	131.08 ± 76.70	0.205
Serum albumin (g/dL)	2.61 ± 0.69	2.60 ± 0.66	2.62 ± 0.73	0.857
Serum sodium (mEq/L)	132.58 ± 6.55	133.06 ± 6.99	131.95 ± 5.93	0.281
Serum creatinine (mg/dL)	1.45 ± 1.25	1.34 ± 0.80	1.60 ± 1.66	0.201
Alanine transaminase (IU/L)	54.49 ± 45.42	53.00 ± 46.98	56.42 ± 43.57	0.630
Mean MELD score	20.35 ± 8.62	19.40 ± 8.26	21.56 ± 8.96	0.109

Among Child-Pugh class A, serum albumin was more likely to decrease in hepatitis B, C-CLD (p=0.089), and serum creatinine was more deranged in the same group (p=0.101). Among the Child-Pugh class B, INR was found more elevated in hepatitis B, C-CLD (p=0.236), and conversely INR was found to be elevated in non-hepatitis B, C-CLD among Child-Pugh class C (p=0.034). Thrombocytopenia was found more severe in Child-Pugh class B of hepatitis B, C-CLD (p=0.032), whereas hyponatremia was found more severe in Child-Pugh class C of non-hepatitis B, C-CLD (p=0.140). Serum creatinine was more deranged in both Child-Pugh classes B (p=0.353) and C (p=0.173) of non-hepatitis B, C-CLD. Lastly, the mean MELD scores were comparable among the two study groups in both Child-Pugh classes A and B, but in Child-Pugh class C it was significantly higher in non-hepatitis B, C-CLD patients as compared to hepatitis B, C-CLD, as shown in Table 3.

**Table 3:** Comparison of biochemical markers in terms of the severity of chronic liver disease. All the p-values were calculated using an independent sample t-test. CLD, chronic liver disease; INR, international normalized ratio; MELD, model for end-stage liver disease

Laboratory investigations	Child-Pugh’s Class	Grouping variables		p-Value
		Hepatitis B, C-CLD (n=94)	Non-hepatitis B, C-CLD (n=73)	
Total bilirubin (mg/dL)	A	0.92 ± 0.64	1.06 ± 0.50	0.563
	B	1.62 ± 1.18	4.27 ± 9.53	0.102
	C	4.95 ± 3.54	5.90 ± 6.04	0.411
Direct bilirubin (mg/dL)	A	0.41 ± 0.24	0.49 ± 0.32	0.489
	B	0.94 ± 0.81	2.22 ± 5.23	0.153
	C	2.78 ± 2.27	3.38 ± 4.02	0.429
Indirect bilirubin (mg/dL)	A	0.51 ± 0.47	0.57 ± 0.23	0.699
	B	0.67 ± 0.42	2.04 ± 4.31	0.063
	C	2.30 ± 1.54	2.51 ± 2.14	0.625
INR	A	1.13 ± 0.21	1.28 ± 0.37	0.182
	B	1.89 ± 2.51	1.35 ± 0.29	0.236
	C	1.92 ± 1.14	2.94 ± 2.43	0.034
Platelet counts (10^9^/L)	A	163.33 ± 98.76	158.40 ± 97.45	0.900
	B	101.44 ± 63.81	136.16 ± 65.11	0.032
	C	104.80 ± 93.08	117.62 ± 79.68	0.538
Serum albumin (g/dL)	A	3.38 ± 0.37	3.70 ± 0.59	0.089
	B	2.67 ± 0.46	2.79 ± 0.39	0.263
	C	2.19 ± 0.59	2.12 ± 0.57	0.624
Serum sodium (mEq/L)	A	136.33 ± 4.49	136.80 ± 1.81	0.757
	B	130.94 ± 5.98	131.70 ± 3.66	0.538
	C	133.50 ± 8.14	130.68 ± 7.65	0.140
Serum creatinine (mg/dL)	A	1.19 ± 0.68	0.82 ± 0.08	0.101
	B	1.37 ± 0.92	1.76 ± 2.31	0.353
	C	1.39 ± 0.74	1.68 ± 1.02	0.173
Alanine transaminase (IU/L)	A	51.22 ± 12.76	43.00 ± 23.93	0.243
	B	57.44 ± 63.22	60.74 ± 56.34	0.824
	C	49.80 ± 39.68	56.43 ± 33.09	0.451
Mean MELD score	A	10.44 ± 4.47	9.60 ± 3.56	0.613
	B	18.72 ± 7.70	18.32 ± 5.84	0.814
	C	24.05 ± 6.42	28.44 ± 6.68	0.006

## Discussion

The major objectives of this study were to evaluate the mortality risk of all CLDs discriminated by etiology and to determine the significance of correlated biological markers. The findings of our endeavor suggest that patients with non-Hepatitis B, C-CLD has a comparatively higher severity of the disease, as measured using Child-Pugh’s classification and MELD score. Secondarily, deranged values of biological markers of disease prognosis and the presence of prior co-morbidities occurred significantly in non-hepatitis B, C-CLD compared to hepatitis B, C-CLD.

The majority of CLD cases in Pakistan are associated with hepatitis B and C [20], and our study further supports this statement as 56.28% of our cohort were serologically positive for hepatitis B or C. This fact is also coherent with previously conducted case series such as in Italy, where hepatitis-positive CLD had a prevalence of 40.1%, and studies conducted in Chicago during 1990, where 58.9% of CLD patients were hepatitis positive. Similar studies in the same population also showed hepatitis C seropositivity in around 40% of CLD cases [21]. A disparity exists in previous literature with regard to the gender distribution of CLD in a given population, as our cohort had a statistically insignificant ratio of 54.5% males and 45.5% females. In previously conducted similar studies, male patients were twice more common than female patients, whereas other studies showed no significant difference in gender distribution [21]. Differences in gender distribution in our study, though insignificant, have shown that contrary to the commonly believed notion that male patients are more prone to contracting CLD due to increased environmental exposure, alcohol consumption, drug abuse, and multiple sexual partners in developing countries like Pakistan, female patients can also be almost equally affected.

Categorizing our findings using Child-Pugh’s classification showed that out of the total hepatitis B, C positive CLD patients, 19% belonged to Child-Pugh class A, 38% to Child-Pugh class B, and 43% to Child-Pugh class C, whereas hepatitis B, C negative CLD patients comprised of 13% Child-Pugh class A, 42% Child-Pugh class B, and 44% Child-Pugh class C. A similar pattern with regard to hepatitis B, C positive CLD patients is found in previously conducted studies, stating that with appropriate treatment, progression to cirrhosis in chronic HCV can even be prevented [22] and can also bring about a reversion of compensated cirrhosis [23]. Early diagnosis, prompt treatment, and increased awareness among a population with regard to viral hepatitis can significantly prevent CLD occurrence. The main application of the Child-Pugh score has been to stratify or to select patients for its CLD-related prognostic analyses. It is a universally applied scoring system used for the past 30 years [24]. However, several important markers of prognosis, disease progression, and mortality risks are not taken into account in this system. In order to bridge this gap and to emphasize the significance of various biological markers found to be important with modern research and practice, this study also incorporated the MELD score for comparing the severity of liver diseases.

The advantages of the MELD score are that its variable inclusion criteria are based on statistical analysis rather than clinical judgment and that the selected variables are objective and are unlikely to be influenced by external factors. Every variable is weighted according to its proper significance on prognosis, and individuals are more precisely scored among large populations [24]. Statistical analysis using the MELD score suggested a significant and directly proportional relationship between the two used scoring systems, as mean MELD scores increased proportionally with Child-Pugh class A to C for both hepatitis B, C-CLD and non-hepatitis B, C-CLD. The values of the MELD score are significantly higher for non-hepatitis B, C-CLD compared to hepatitis B, C-CLD, further suggesting that regardless of the scoring system used, mortality risk is significantly higher in non-viral etiologies of CLD.

The biochemical markers for severity assessment in CLD patients showed that total bilirubin, serum creatinine, and INR had higher values in Non-hepatitis B, C-CLD group. Hyponatremia was also found more frequently in the same group. Surprisingly thrombocytopenia was more pronounced in hepatitis B, C-CLD. Serum albumin level below (3.5 g/dL) is a known predictor of CLD and decreased survival [25]. In the data collected, serum albumin levels decreased proportionately in accordance with Child-Pugh’s classification and MELD score. Patients in non-hepatitis B, C-CLD group had lower serum albumin levels, similar to that in hepatitis B, C-CLD group.

Increased mortality rates among non-hepatitis B, C-CLD was previously studied in the same population [20]. Another study conducted in a similar pattern showed a significantly higher age of hepatitis C positive group as compared to seronegative CLD, and our study had a similar mean age in both the groups [25]. In the same study, seronegative CLD was more prevalent in males, whereas seropositive CLD was more prevalent in females [25], another finding contrasting our study which had equal gender predisposition in all the groups. ALT was slightly higher in seropositive CLD [25], while our study showed slightly higher ALT among seropositive CLD in Child-Pugh class A and vice versa in Child-Pugh classes B and C. Serum albumin was quite higher than that in our study, although equally deranged in both groups, similar to our findings. Platelet counts were more affected in non-viral CLD, contrasting to our results [25]. In the conclusion of that study, hepatic decompensation and mortality were found indifferent in both the groups, whereas our study stated increased severity markers among non-viral CLD.

Platelet counts were found decreased in viral CLD by two different studies similar to our findings [26,27]. Serum albumin was found more decreased in HCV CLD in one study [26], whereas it was equally decreased in both etiologies according to the other research [27]. ALT was found higher in HCV-CLD in both the studies, opposing our results [26,27]. Total bilirubin was higher in non-viral CLD according to one study [26], synchronizing with our findings, whereas the other study opposed our results by revealing higher bilirubin in viral CLD [27]. A higher age group was found in viral CLD, whereas the male gender was prevalent in non-viral CLD [27]. A higher Child-Pugh score was concluded by the above study in non-viral CLD, similar to our results [27]. Comparing the Child-Pugh classes, the higher frequency comprised Child-Pugh class B in non-hepatitis B, C-CLD, whereas a similar frequency of Child-Pugh class C was observed in both the study groups [27], a finding similar to our study results, although our report suggested higher frequencies in Child-Pugh class C of both the study groups compared to them.

The limitations of our study included a retrospective study design along with a relatively smaller sample size in a single-center study, hence not being able to generalize the results over a large population suffering from CLD.

## Conclusions

Non-hepatitis B, C-CLD was proved to be milder in Child-Pugh class A as compared to hepatitis B, C-CLD, but its mortality risk increased with severity, as mean MELD score was found significantly higher in Child-Pugh class C. Furthermore, several biochemical markers and co-morbidities correlated more with non-hepatitis B, C-CLD as compared to hepatitis B, C-CLD. In conclusion, our research was able to identify possible biochemical markers and significant etiologies of all patients suffering from CLD.
